# Genome-Wide Screen in *Saccharomyces cerevisiae* Identifies Vacuolar Protein Sorting, Autophagy, Biosynthetic, and tRNA Methylation Genes Involved in Life Span Regulation

**DOI:** 10.1371/journal.pgen.1001024

**Published:** 2010-07-15

**Authors:** Paola Fabrizio, Shawn Hoon, Mehrnaz Shamalnasab, Abdulaye Galbani, Min Wei, Guri Giaever, Corey Nislow, Valter D. Longo

**Affiliations:** 1 Andrus Gerontology Center and Department of Biological Sciences, University of Southern California, Los Angeles, California, United States of America; 2 Laboratoire de Biologie Moléculaire de la Cellule, Centre National de la Recherche Scientifique, Ecole Normale Supérieure de Lyon, Université de Lyon, Lyon, France; 3 Department of Genetics, Stanford University, Palo Alto, California, United States of America; 4 Department of Pharmaceutical Sciences, University of Toronto, Toronto, Ontario, Canada; 5 Department of Molecular Genetics, University of Toronto, Toronto, Ontario, Canada; Stanford University Medical Center, United States of America

## Abstract

The study of the chronological life span of *Saccharomyces cerevisiae*, which measures the survival of populations of non-dividing yeast, has resulted in the identification of homologous genes and pathways that promote aging in organisms ranging from yeast to mammals. Using a competitive genome-wide approach, we performed a screen of a complete set of approximately 4,800 viable deletion mutants to identify genes that either increase or decrease chronological life span. Half of the putative short-/long-lived mutants retested from the primary screen were confirmed, demonstrating the utility of our approach. Deletion of genes involved in vacuolar protein sorting, autophagy, and mitochondrial function shortened life span, confirming that respiration and degradation processes are essential for long-term survival. Among the genes whose deletion significantly extended life span are *ACB1*, *CKA2*, and *TRM9*, implicated in fatty acid transport and biosynthesis, cell signaling, and tRNA methylation, respectively. Deletion of these genes conferred heat-shock resistance, supporting the link between life span extension and cellular protection observed in several model organisms. The high degree of conservation of these novel yeast longevity determinants in other species raises the possibility that their role in senescence might be conserved.

## Author Summary

Model organisms have been instrumental in uncovering genes that function to control life span and to identify the molecular pathways whose role in aging is conserved between the evolutionarily distant unicellular yeast and mice. Because yeast are particularly amenable to genetics and genomics studies, they have been used widely as model system for aging research. Here we have exploited a powerful genomic tool, the yeast deletion collection, to screen a pool of non-essential deletion mutants (∼4,800) to identify novel genes involved in the regulation of yeast chronological life span. Our results show that normal life span depends on functional mitochondria and on the cell's ability to degrade cellular components and proteins by autophagy. Our data indicate that a cell signaling protein, CK2, and diverse cellular processes such as fatty acid metabolism, amino acid biosynthesis, and tRNA modification modulate yeast chronological aging. The high level of conservation of the novel life span regulatory genes uncovered in this study suggests that their role in longevity regulation might be conserved in higher eukaryotes.

## Introduction

Yeast, worms, and flies have been studied extensively to identify the genetic determinants of aging. Studies conducted in these model organisms have demonstrated a partially conserved life span regulatory role for the nutrient-sensing/insulin/IGF-I-like pathways, which are found in species ranging from yeast to mice [1], [2]. Two different paradigms have been established to study the life span of yeast. Chronological life span (CLS) measures the mean and maximum survival time of populations of non-dividing yeast [3], while replicative life span (RLS) refers to the number of daughter cells generated by an individual mother cell before it ceases to divide [4], [5]. Several genes similarly affect both CLS and RLS, while others have opposite effects on the two aging paradigms, suggesting that the mechanisms underlying the CLS and RLS are only partially overlapping [6], [7].

By screening transposon-mutagenized yeast populations (previously selected for their ability to withstand either oxidative or heat stress) for mutants with an extended CLS, the serine-threonine protein kinase Sch9 and adenylate cyclase (Cyr1) were identified as negative regulators of longevity [8]. The effect of the Ras/Cyr1/PKA pathway on aging had been previously described based on its role in glucose signaling [9],[10]. Reducing the activity of Sch9 or Cyr1 and consequently that of the nutrient-sensing pathways they participate in (TOR/Sch9 and Ras/Cyr1/PKA), CLS is extended by up to 3-fold, with a concomitant increase in resistance to cellular stress [8]. Consistent with this observation, inactivation of the G-protein Ras2, which promotes Cyr1 function, also extends CLS [11]. The two closest metazoan homologues of Sch9, Akt and S6K, have been implicated in the insulin/IGF-I-like signaling and life span regulation in all the major model organisms [1], [12], [13], [14]. Conversely, the role of the Ras/Cyr1/PKA signaling in aging of higher eukaryotes has been more elusive [15]. However, recently, mice lacking adenylate cyclase 5 (AC5) have been reported to be long-lived and fibroblasts derived from these mice have been shown to be resistant to oxidative stress, consistently with previous observations in yeast *cyr1* mutants [16]. Moreover, the disruption of RIIβ, which codes one of the mammalian PKA regulatory subunits, has been shown to promote median and maximum life span extension in male mice [17].

In the last few years several laboratories have turned to the yeast CLS to elucidate how post-mitotic and reversibly arrested cells age in higher eukaryotes. However, some concern over the extensibility of this model has been raised in light of recent observations that acetic acid, which accumulates extracellularly in the culture medium, is a key cause of chronological aging in yeast [18]. The question is if acetic acid-dependent cell death is relevant to aging in metazoans. Previously, we found that ethanol accumulates during chronological aging and promotes death, and that its removal extends CLS [7]. We also found that glycerol replaces ethanol in cultures of long-lived yeast and its synthesis is crucial for longevity extension [19]. Burtner et al. have proposed that ethanol is metabolized to produce acetic acid, to which long-lived mutants are more resistant than wild type yeast [18]. Others have suggested that ethanol removal via the activation of gluconeogenesis mediates longevity extension [20]. Although ethanol and acetic acid at high concentrations may in fact be directly toxic to the cell, for *S. cerevisiae* they are commonly encountered carbon sources and thus, their removal may extend life span in part by promoting calorie restriction, a non-genetic intervention known to extend the life span of a broad range of species [21]. Further studies are needed to clarify the range of metabolic changes that occur during chronological aging to understand how acetic acid or other acids, ethanol, or glycerol might be relevant to aging of multicellular eukaryotes. While it is plausible that, by analogy with yeast, the composition of the extracellular milieu of multicellular organisms contributes to aging [22], different metabolites might be implicated in aging of multicellular species. Notably, mutations in the Sch9 and Ras/Cyr1/PKA pathways in yeast extend CLS even after removal of extracellular carbon sources indicating that the release of ethanol and acetic acid into the medium is not a requirement for these genes to exert their effect on longevity [23].

Previously, Powers and coworkers used the yeast diploid homozygous deletion collection, which covers 96% of the yeast genome [24], [25], to develop an assay to monitor the CLS of all individual deletion mutants. The principal finding of their screen was the identification of the TOR pathway as a pro-chronological aging pathway. In fact, deletion of either *TOR1* or of several other genes controlled by the TOR cascade, e.g. *GLN3* (encoding a transcription factor induced by the amino acid starvation response), prolongs CLS [26]. A pro-chronological aging role for the serine/threonine kinase Tor1 has recently been confirmed by others [27] and the down-regulation of the TOR signaling cascade has also been implicated in the CLS extension induced by calorie restriction [23]. In yeast, Sch9 is a direct target of the Tor-containing complex 1 (TORC1) and its inactivation mediates the CLS extension observed in a *tor1Δ* context [23], [28], [29]. A role for TOR in longevity regulation has been confirmed in worms and flies [12], [30], [31] and recently, by analogy with yeast [26], mice and flies treated with rapamycin, an inhibitor of TORC1, have been reported to live longer than untreated controls [32], [33]. The conservation of the TOR kinases and of their role in aging across species suggests that rapamycin may represent the first drug that functions to prolong life span of multiple species including mammals.

High rates of false positives and negatives are common in genomic screens [34], accordingly, we decided to use a different methodological approach to screen for gene deletions that affect CLS. We relied on competitive screening of pools of the ∼4800 non- essential deletion mutants in the haploid wild type BY4741 genetic background [35]. Notably, the deletion strategy designed to construct the yeast knock-out collection generates two unique 20bp DNA tags on each deletion mutant (uptag and downtag). These tags allow the monitoring of the changes in representation of each deletion mutant in a pool using a barcode microarray that carries the complement of the tag sequences. Thus, our method differs from that of Powers et al. in that: 1) it measures the CLS of pooled, competitive cultures of standard size (50 mL) instead of that of individual micro-cultures (0.2 mL) of each deletion mutant, and 2) it employs a DNA microarray-based technique to quantify the age-dependent individual strain abundance rather than absorbance measurement of individual cultures.

## Results

### Identification of CLS regulatory genes by K-means clustering analysis

**Figure 1:**
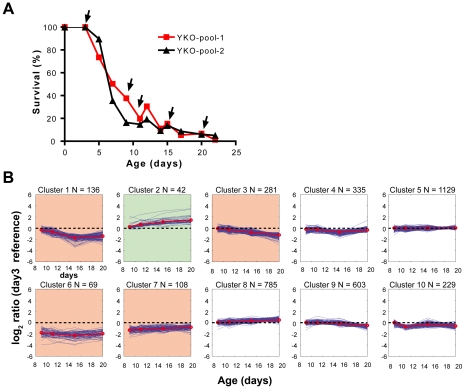
Screen of the yeast homozygous deletion collection for life span regulatory genes. (A) CLS of the two pools used for the yeast deletion collection screen. The black arrows indicate when cell samples were collected for DNA extraction. The experiment was conducted by incubating the yeast pools in SDC medium throughout the experiment. (B) 10 aging profile clusters derived by K-means clustering analysis. The y-axis displays the log_2_ fold ratio of tag intensity for each strain at each time point relative to the day 3 time point. Plots highlighted in red represent clusters classified as short-lived and the plot highlighted in green represents a cluster classified as long-lived. The dashed black line demarcates the boundary between short and long-lived strains. The red line is the centroid (average profile) for each cluster.

**Figure 2:**
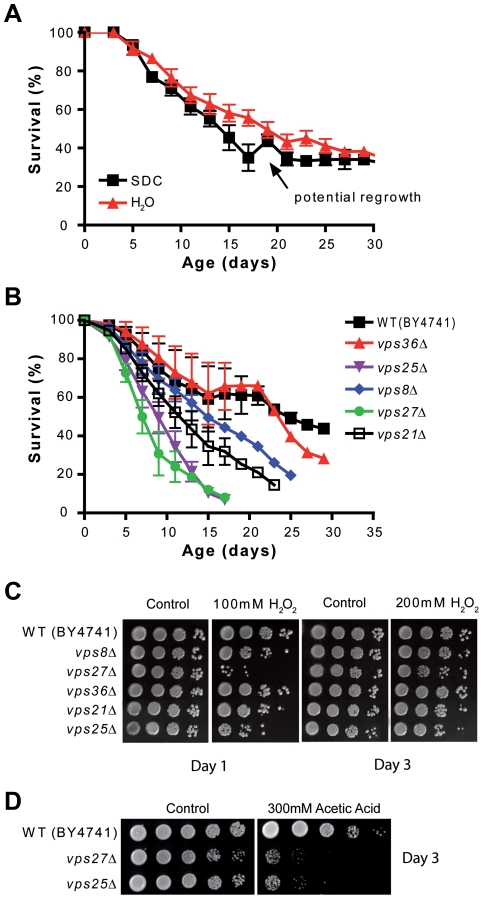
*VPS* genes are required for starvation/extreme CR–dependent life span extension and resistance to oxidative stress and acetic acid. (A) CLS of wild type (BY4741) in SDC medium and under starvation/extreme CR. Starvation/extreme CR was obtained by transferring the yeast culture to water at day 3 (see Materials and Methods). The arrow indicates the time at which adaptive regrowth might be occurring. (B) CLS of wild type (BY4741) and of the *vps36Δ*, *vps25Δ*, *vps8Δ*, *vps27Δ*, and *vps21Δ* deletion mutants. Yeast cultures were transferred from medium to water at day 3. Data show mean±SEM of three experiments. (C) Resistance to H_2_O_2_ of the same mutants. After a 30 minute-exposure to 100–200 mM H_2_O_2_ in K-buffer, day 1–3 cells from SDC cultures were serially diluted and plated on YPD plates. D) Resistance to acetic acid of wild type cells, *vps25Δ* and *vps27Δ* mutants. Day 3 cultures were exposed to 300 mM of acetic acid for 3 hours before being serially diluted and plated onto YPD plates.

In order to identify novel genes implicated in life span regulation we measured the CLS of two independent yeast populations obtained by diluting two identical pools of 4×10^6^ frozen cells into 50 mL of synthetic complete medium containing 2% glucose (SDC). After 3 days, the two yeast cultures reached a densitiy of 1.5×10^8^/mL. Because in a standard CLS experiment, no further increase of cell density is usually observed after 3 days, the number of colony forming units (CFUs) measured at day 3 was defined as 100% survival [3]. The survival curves for each pooled culture are shown in Figure 1A, the actual CFUs data are reported on Table S1. Interestingly, both mean and maximum survival times were significantly shorter as compared to those of the wild type BY4741 (Figure 2A) [7]. This may be due to the fact that numerous deletions reduce survival [36] and/or the survival defects of the corresponding mutants are exacerbated when they grow in the presence of 4800 other deletion strains. Consistent with this hypothesis, we observed a high number of budded cells in pooled cultures (data not shown) suggesting that several deletions may cause an increase of the non-quiescent fraction of cells [37]. Notably, post-diauxic and stationary phase cultures of yeast aging chronologically are composed of both quiescent and non-quiescent cells, although cell division within the population grown in SDC medium appears to be minimal and to not affect the measurement of CLS [38], [39]. Non-quiescent cells differ from quiescent cells in that they do not arrest in G_0_ properly, are more susceptible to reactive oxygen species and apoptosis, and lose viability more rapidly than G_0_-arrested quiescent cells [38], [40]. The survival curves of both pooled cultures showed an increase of CFUs at day 12 and 15 (Figure 1A). This may be caused by specific mutants that can utilize the low nutrient medium for growth (see next section, paragraph on adaptive regrowth) [41].

To measure the viable cells corresponding to each individual mutant, aliquots containing 6.25×10^5^ cells of each culture were diluted in fresh medium and grown until they reached a cell density of 10^7^/mL. Samples corresponding to approximately 2×10^7^ cells were frozen at day 3, 9, 11, 15, and 20. Genomic DNA was extracted from cell pellets as described by Pierce et al. [35]. Aging cultures were not used directly for DNA extraction to avoid any noise that might be contributed by unlysed dead cells. Both uptags and downtags were PCR-amplified and hybridized to Affymetrix TAG4 arrays, which were processed as previously described [35]. For each time point, the log_2_ intensity ratio was calculated with respect to day 3 (100% survival) and the aging profiles for each individual mutant were extracted (Table S2). The root squared mean errors (RSME) between the two replicates were calculated and mutants with high RSME (90^th^ percentile) were excluded from the analysis (Table S2). The microarray results were used to approximate a survival curve for each individual deletion strain by multiplying the fold ratio change in the microarray results by the CFUs relative to the pools (Table S3). K-means clustering analysis (K = 10) was performed on the averaged log_2_ ratios between the two pools and five clusters corresponding to mutants whose life span trajectories differed from that of the mean of the pool were identified by manual inspection (Figure 1B). While mutants belonging to four clusters, 1-3-6-7, were classified as short-lived, cluster 2 included long-lived mutants (Figure 1B, Tables S5, S6). In parallel, we also used a significance analysis of time course microarray experiments developed for identifying differentially expressed genes in a time course to test for consistency between our replicates (EDGE analysis, see Materials and Methods, Tables S4, S5, S6) [42], [43].

### Short-lived mutants

K-means clustering indicated that 594 genes are required for normal life span (Table S5). Among these, we observed an enrichment of genes belonging to the “mitochondrion” gene ontology group (GO: 0005739, 24.3% *vs* 15.4%, relative *vs* background frequency), with 6.1% and 3.4% being part of the “mitochondrial inner membrane” (GO:0005743, background frequency 2.4%) and “mitochondrion degradation” (GO: 0000422, background frequency 0.5%) GO categories, respectively (Table S7). Many mitochondrial genes among those whose deletion shortens the CLS were expected because functional mitochondria are important for survival after the diauxic shift when glucose is depleted and yeast switch from fermentative to respiratory metabolism [44], [45].

The list of genes whose deletion is associated with reduced life span is also enriched in members of the “autophagy”, “macroautophagy”, and “microautophagy” GO biological process categories (GO: 0006914, GO:0016236, GO: 0016237, respectively) (Table S7). This suggests that protein and organelle turnover by vacuolar digestion is required for normal survival and may contribute to prolong yeast life span, consistently with proposals for *C. elegans* and *Drosophila* [46], [47], [48]. Among the shortest-lived mutants, we identified several mutants carrying deletions of genes implicated in protein targeting to the vacuole (*VPS* genes). To validate our screening results we measured the life span of mutants lacking individual Vps proteins, namely Vps25, Vps27, Vps21, Vps36, and Vps8 (q<0.1, EDGE analysis, Table S5). Four of the five mutants were short-lived (Figure 2B, see below).

All the experiments described hereafter in the BY4741 background were performed by switching the cells to water at day 3 after the yeast populations had reached saturation rather than by leaving them in medium. Incubation in water represents a form of starvation/extreme calorie restriction (CR), which, similarly to the reduction of glucose content in the growth medium, promotes life span extension [7], [39], [44], [49]. Previously, we have shown that similar pathways are implicated in both starvation (water)- and CR (0.5% glucose)-dependent CLS extension [23]. We have also shown that virtually all the mutants that show longevity extension in SDC are long-lived also when different media are used for the survival studies (e.g. synthetic complete + 0.5% glucose, water, or SDC without tryptophan on plates) ([7], [19], [23] and M. Wei, unpublished results). The monitoring of longevity in water is also a useful means to rule out any occurrence of adaptive regrowth, which can confound the interpretation of our survival data. Adaptive regrowth occurs when aging cells acquire mutations that allow them to reenter the cell cycle in conditions than normally do not promote growth [41]. It is usually observed in wild type yeast after a large fraction of the yeast population is inviable, because it depends upon the nutrients released by the dead cells to occur and can be prevented by switching the cells to water and washing them periodically [41], [44]. The frequency of adaptive regrowth is increased in mutants that are more sensitive to DNA damage, e.g. *sod1Δ* or *sgs1Δ* [39], [41]. Since BY4741 shows a modest response to starvation/extreme CR in comparison with other genetic backgrounds (Figure 2A) ([7], [39] and P. Fabrizio unpublished results), we hypothesized that this may depend in part on a tendency of BY4741, in contrast with other strains, to resume cell division when a large fraction of cells is still alive. Thus, to obtain more conclusive data relative to the nature of our putative BY4741 short- and long-lived mutants, we performed our survival assays in water. These experiments test the role of the putative life span regulatory genes in starvation/extreme CR-dependent life span extension and do not represent a direct validation of our screen, which did not assay for survival in water. Nevertheless, the individual strain survival assays in water allow us to identify mutations that diminish or prolong life span in the BY4741 background and to avoid mistaking deletions that promote adaptive regrowth for those that extend life span.

Deletion of *VPS25* and *VPS27* causes a dramatic reduction of life span (average of three independent experiments) to a level below that of wild type cells in SDC (Figure 2A and 2B) (p<0.001). Lack of Vps21 and Vps8 reduced life span under starvation conditions to a level similar to that of wild type cells incubated in SDC (p<0.01 and 0.05, respectively) (Figure 2A and 2B). In contrast, the *vps36* deletion mutant lived as long as the wild type BY4741. Thus, Vps36 is not required for the starvation/extreme CR-dependent life span extension (Figure 2B).

Since the Vps proteins are important for protein degradation, they may contribute to the removal of oxidized/damaged proteins known to accumulate during aging [50], [51]. Consequently, in their absence yeast might be more sensitive to oxidants. To test this hypothesis, we monitored the resistance to hydrogen peroxide (100–200 mM for 30 minutes) of different *vps* mutants during chronological aging at day 1 and 3 and found an association between life span and resistance to oxidative stress, with *vps25Δ* and *vps27Δ* being the shortest-lived and also the most stress sensitive and *vps36Δ* having a normal life span and also unaltered stress resistance (Figure 2C). The *vps25Δ* and *vps27Δ* mutants were also tested for their resistance to acetic acid by exposing day 3 cultures to 300 mM acetic acid for 3 hours. Both mutants showed an increased sensitivity to acetic acid in comparison with the wild type (Figure 2D). Combined with the increased sensitivity to hydrogen peroxide, this appears to reflect a general susceptibility of these mutants to stress and not the mechanism leading to early cell death, since 300 mM acetic acid is much higher than the level normally encountered/generated by cells (Figure 2D) [18]. Together, these results indicate that functional Vps-dependent protein degradation systems are essential for starvation-dependent life span extension.

### Long-lived mutants

**Figure 3:**
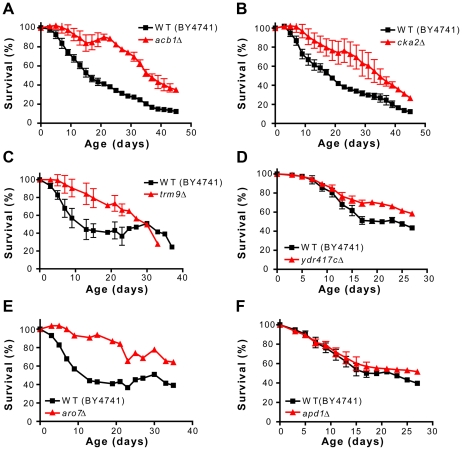
Novel long-lived mutants. CLS of cultures under starvation/extreme CR of wild type (BY4741) and (A) *acb1Δ*, (B) *cka2Δ*, (C) *trm9Δ*, (D) *ydr41cΔ*, (E) *aro7Δ*, and (F) *apd1Δ* mutants. All figures show an average of 2–3 experiments except (F), which shows a representative experiment. The CFUs at day 3 before cells were transferred to water were: 131.3±5.1, 129.8±7.2, 111±8, 77.1±8.1, 169±2, 48.1±4.8, 152.1±3.3 (cells ×10^6^/mL±SEM) for wild type, *acb1 Δ*, *cka2 Δ*, *trm9Δ*, *ydr41cΔ*, *aro7Δ*, and *apd1Δ*, respectively.

**Figure 4:**
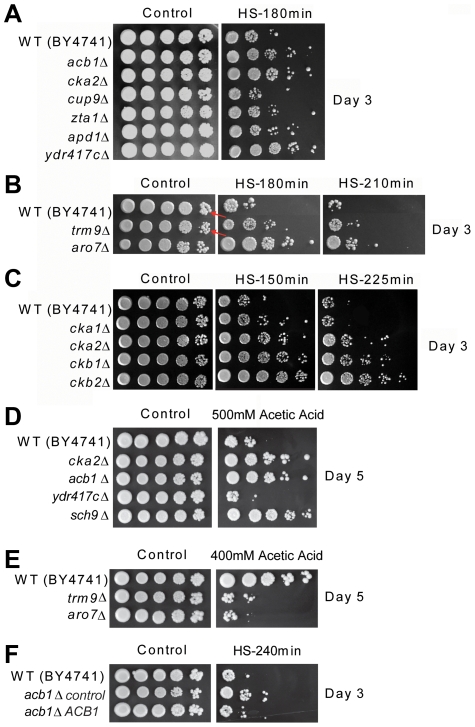
Long-lived mutants are resistant to heat. Day 3 chronologically aging wild type (BY4741) and the following mutants were serially diluted, plated onto YPD plates and heat-shocked at 55°C for 150–225 minutes: (A) *acb1Δ*, *cka2Δ*, *cup9Δ*, *zta1Δ*, *apd1Δ*, *ydr417c Δ*, (B) *trm9Δ* and *aro7Δ*; in the control panel (highest dilution factor) the arrows indicate the size of the *trm9Δ* colonies (row 2) in comparison with that of the wild type ones (row 1), and (C) *cka1Δ*, *cka2Δ*, *ckb1Δ*, *ckb2Δ*. (D) Day 5 cultures of wild type, *acb1Δ*, *cka2Δ*, *ydr417cΔ*, *and sch9Δ* were exposed to 500 mM acetic acid for 180 minutes, serially diluted, and plated onto YPD plates. (E) Day 5 cultures of wild type, *trm9Δ*, and *aro7Δ* exposed to 400 mM acetic acid for 180 minutes. (F) Day 3 cultures of wild type and *acb1Δ* transformed with either a centromeric plasmid carrying a wild type *ACB1* gene or a control vector were serially diluted, plated onto YPD plates, and heat-shocked at 55°C for 240 minutes. The cultures used for the stress resistance experiments were in either SDC (wild type) or SDC-uracil (*acb1Δ*).

While mutations that shorten life span may not be directly associated with aging but rather may simply cause reduced cellular fitness, mutations that extend life span are, in most cases, indicative of an involvement of the corresponding genes in the aging process. K-means clustering analysis allowed us to identify 42 putative long-lived mutants (Table S6). To select the strains to be retested for longevity under starvation/extreme CR, after excluding the mutants carrying deletions of dubious ORFs not overlapping any ORF/gene on the complementary strand (*YOR012W*, *YDR102C*) and the *ydr442wΔ* and *sfl1Δ* mutants, which showed a marked flocculation phenotype in synthetic medium, we randomly chose 14/42 mutants (Table 1). Five of them, *acb1Δ*, *cka2Δ*, *trm9Δ*, *ydr417cΔ*, and *aro7Δ* were confirmed as long-lived in the BY4741 genetic background (Figure 3A–3E, p<0.01–0.05). The life span of mutants lacking either Cup9, Apd1, Zta1, or Ssn2, a transcriptional repressor, a protein required for normal localization of actin patches, a quinone reductase, and a subunit of the RNA polymerase II mediator complex, respectively, was not significantly different from that of the wild type (Figure 3F, Figure S1, and data not shown). While the mutants living significantly longer than the wild type (*acb1Δ*, *cka2Δ*, *trm9Δ*, *ydr417cΔ*, and *aro7Δ*) were heat resistant (Figure 4A and 4B) (see below) no major changes in heat-shock resistance were observed in the mutants (*cup9Δ*, *apd1Δ*, *zta1Δ*, *ssn2Δ*) whose life span extension was not significant (Figure 4A and data not shown).

**Table 1:** Putative long-lived deletion mutants selected to confirm their long-lived phenotype.^1^

Gene	p-value	q-value
*BUL1*	9.69E-06	0.009297
*CKA2*	0.000446	0.021931
*CUP9*	0.00101	0.025109
*PPG1*	0.00139	0.025842
*ZTA1*	0.001574	0.02719
*PAN2*	0.003523	0.032853
*FAR3*	0.005765	0.037948
*APD1*	0.006201	0.037948
*SSN2*	0.00631	0.037956
*FAR11*	0.015981	0.05204
*ACB1*	0.031109	0.070276
*ARO7*	0.033036	0.072101
*YDR417C*	0.187961	0.200623
*TRM9*	0.296211	0.264509

^**1**^p and q values were obtained by EDGE analysis performed on the two biological replicates (see Materials and Methods).

The deletion of *ACB1*, which encodes a highly conserved acyl-CoA-binding protein implicated in acyl-CoA-ester transport, sphingolipid synthesis, and fatty acid chain elongation [52], caused a 2.2-fold mean life span extension in the genetic background BY4741 (Figure 3A). Lack of Acb1 also increased heat-shock resistance in chronologically aging cells (Figure 4A), a phenotype observed in the great majority of long-lived mutants so far identified [53]. Similarly, resistance to a very high concentration of acetic acid was enhanced by the deletion of *ACB1* (Figure 4D). However, in contrast with other long-lived yeast, the *acb1Δ* mutants did not exhibit any resistance to oxidative stress measured as the ability to maintain viability after 30 minute-treatment with 200–300 mM H_2_O_2_ (data not shown). To test the role of Acb1 in life span regulation in non-CR conditions (incubation in SDC medium) and in different genetic backgrounds, we deleted *ACB1* in W303-1A and DBY746, which usually undergo adaptive regrowth only in the late phases of chronological survival ([23], [44] and P. Fabrizio unpublished results). In these backgrounds the *acb1Δ* mutants showed severe growth defects, were slightly short-lived and heat-shock sensitive (data not shown). Since a yet uncharacterized adaptation that leads to faster growth has been reported to occur at high frequency in *acb1Δ* cultures [54], we verified the linkage between *ACB1* and our phenotypes of interest in the BY4741 *acb1Δ* mutant from the deletion collection, which displays only a modest growth defect and might carry suppressor mutations. To do this, the mutant was transformed with a centromeric plasmid containing the *ACB1* gene under its own promoter and both heat-shock resistance and CLS were monitored. *ACB1* expression complemented both heat-shock resistance and life span extension of the *acb1Δ* mutant (Figure 4F and Figure S2) indicating that both phenotypes are caused by the deletion of *ACB1*.

**Figure 5:**
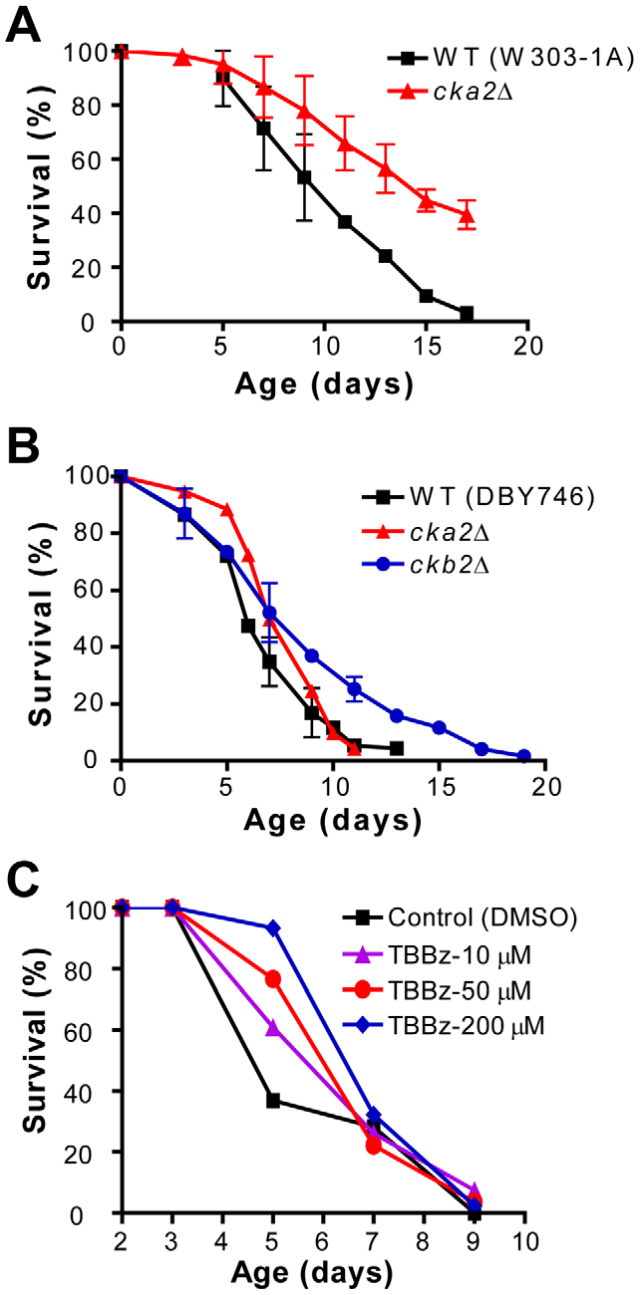
CK2 regulates life span in different genetic backgrounds. (A) CLS of wild type (W303-1A) and *cka2Δ*. (B) CLS of DBY746 and of mutants lacking either Cka2 or Ckb2. (C) CLS of wild type DBY746 treated with increasing concentrations of TBBz (10–200µM) at day 2 and 5. DMSO was used as a vehicle. (A,B) show an average of 2–3 experiments. A representative experiment is shown in (C). All the survival studies were performed leaving the yeast cultures in medium until the end of the experiment.

**Figure 6:**
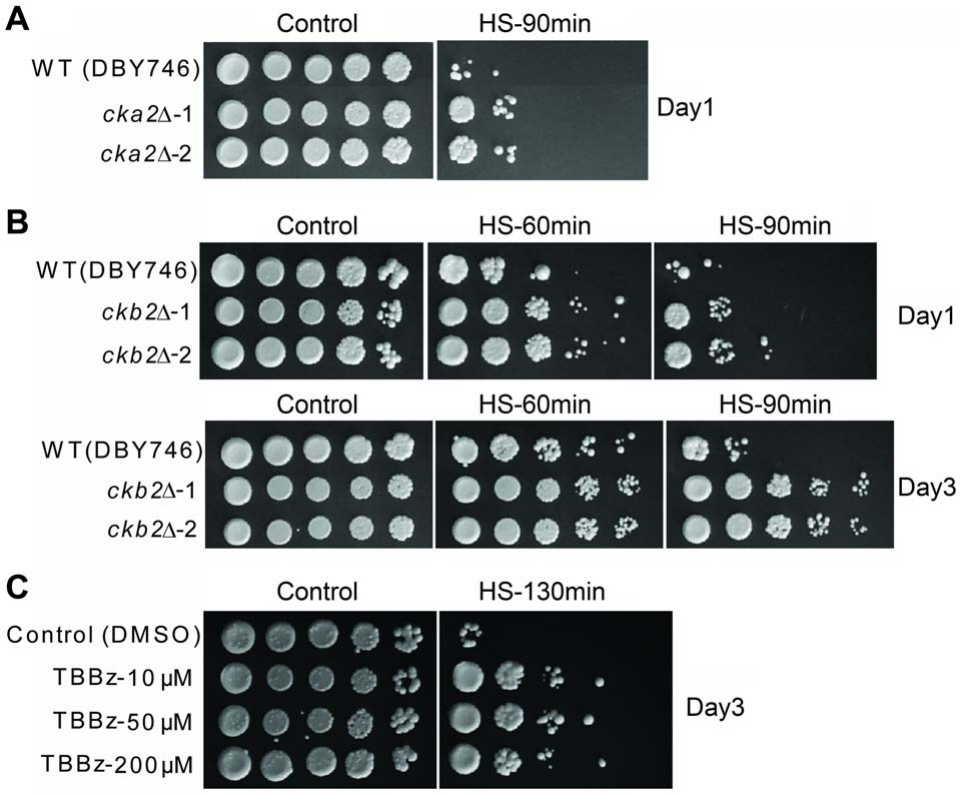
Role of CK2 in the regulation of heat-shock resistance. (A) Heat-shock resistance of two different *cka2Δ* isolates generated in the DBY746 genetic background monitored at day 1. (B) Heat-shock resistance of DBY746 and of two different cultures of the *ckb2Δ* mutant at day 1–3. (C) Heat-shock resistance of DBY746 yeast treated with 10, 50, 200 µM of TBBz at day 2.

The deletion of *CKA2*, which encodes one of the two catalytic subunits of the serine-threonine kinase CK2, approximately doubled the mean life span of BY4741 under starvation/extreme CR (Figure 3B). CK2 is a tetramer comprised of two catalytic and two regulatory subunits, which regulates cell growth/division (among other functions) in all eukaryotes so far investigated [55], [56]. Analogous to the *acb1Δ* mutant, yeast lacking Cka2 are heat-shock and acetic acid resistant but not resistant to H_2_O_2_ (Figure 4A and 4D, and data not shown). The deletion mutants corresponding to either one of the two regulatory subunits (Ckb1 and Ckb2) were also resistant to heat. Conversely, yeast lacking the catalytic subunit Cka1 were approximately as resistant as wild type cells (Figure 4C). These results suggest that the activity of the holoenzyme and not only of the free catalytic subunits, which are known to have functions independent of the regulatory subunits [57], are responsible for the phenotypes observed. Furthermore, the involvement of both Ckb1 and Ckb2 in the regulation of stress resistance is in agreement with the requirement of both regulatory subunits for the full CK2 activation [56]. The role of CK2 in life span regulation and heat-shock resistance was confirmed in SDC medium in the W303-1A and DBY746 genetic backgrounds (Figure 5A and 5B, Figure 6A). To support the hypothesis that the holoenzyme activity promotes aging, we deleted *CKB2* in DBY746 and monitored life span and stress resistance of the corresponding mutant. Lack of Ckb2 promoted a modest but significant (p<0.05) longevity extension and a marked increase of heat resistance in comparison with the wild type (Figure 5B, Figure 6B). Two highly specific CK2 inhibitors, 4,5,6,7-tetrabromo-benzotriazole (TBBt) and 4,5,6,7-tetrabromo-benzimidazole (TBBz), have been identified and shown to inhibit the activity of the holoenzyme [57]. More specifically, in yeast TBBz inhibits the CK2 complex selectively and not the free Cka2 catalytic subunits [57]. We tested both inhibitors in our system and found that TBBz (10–200 µM) but not TBBt (5–15 µM) increased substantially the heat resistance of day 3 DBY746 cultures (Figure 6C, Figure S3A). Furthermore, TBBz but not TBBt improved survival at day 5 (Figure 5C, Figure S3B). Together, these results confirm that the activity of the holoenzyme is responsible for the pro-aging effect of Cka2.

**Figure 7:**
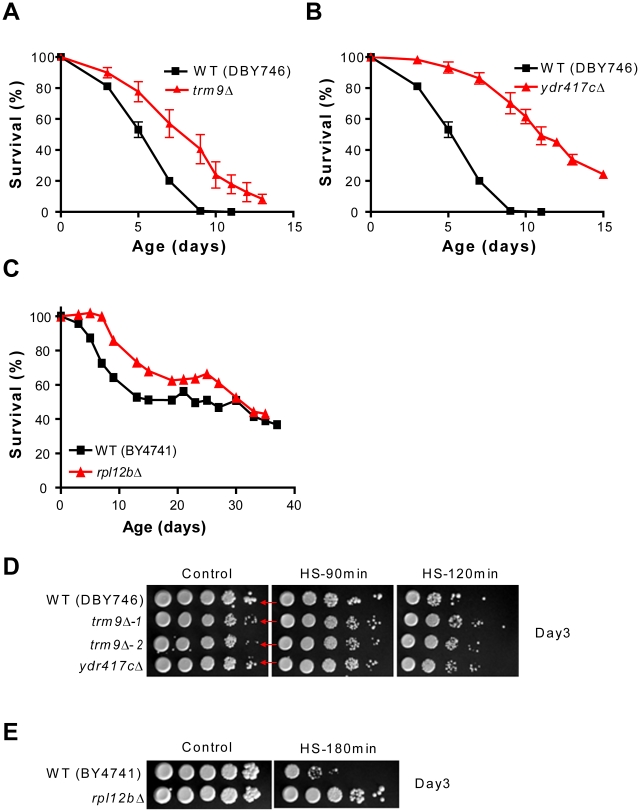
Role of *TRM9* and *YDR417C* in life span regulation and heat resistance. CLS of DBY746 and mutants lacking either (A) Trm9, or (B) Ydr417c. (C) CLS of BY4741 and *rpl12bΔ*. (D) Day 3 heat-resistance of two different isolates of the *trm9Δ* mutant generated in the DBY746 background and of a *ydr417cΔ* mutant. In the control panel (highest dilution factor) the arrows indicate the colony size of the long-lived *trm9Δ* and *ydr417cΔ* mutants (row 2–4) as compared to that of the wild type (row 1). (E) Heat-resistance of BY4741 and of a mutant lacking Rpl12b at day 3. The survival studies in (A,B) were conducted incubating the cultures in SDC medium until the end of the experiment. The data shown represent an average of 2–3 experiments. Yeast cultures in (C) were transferred to water at day 3 (see Materials and Methods). A representative experiment is shown.

*TRM9* codes a tRNA methyltransferase that methylates uridine residues at the wobble position in tRNA(Glu) and tRNA(Arg3) [58]. Its deletion in BY4741 almost tripled yeast mean CLS under starvation/extreme CR (Figure 3C), increased heat resistance (Figure 4B), but reduced resistance to acetic acid (Figure 4E). Similar results were obtained by testing a *trm9Δ* mutant generated in the DBY746 background in SDC medium (Figure 7A and 7D). In this background, lack of Trm9 exacerbated the mild growth defect observed in BY4741 as estimated by colony size (Figure 4B, Figure 7D).

The deletion of *YDR417C* also promoted longevity extension and heat-shock resistance but reduced acetic acid resistance (Figure 3D, Figure 4A and 4D). This dubious ORF overlaps widely with the gene coding the ribosomal protein Rpl12b. The life span and resistance to heat of yeast lacking Rpl12b were similar to that of the *ydr417cΔ* mutant (Figure 7C and 7E, Figure 4A, Figure 3D). Notably, no protein expression corresponding to *YDR417C* was detected by analyzing strains carrying either the GFP- or TAP-tagged version of this ORF. By contrast, Rpl12b was detected using both tagging systems [59], [60]. In DBY746 the deletion of *YDR417C* caused a marked reduction of colony size, almost doubled the mean life span in SDC medium (p<0.005), and increased heat-shock resistance of chronologically aging yeast (Figure 7B and 7D).

*ARO7* encodes for chorismate mutase, which is required for the biosynthesis of the aromatic amino acids tyrosine and phenylalanine from chorismate. Its deletion lowered fermentative growth rates (data not shown) and caused a ∼60% reduction of the total number of cell counted at day 3. Approximately 5×10^7^cells/mL were alive at day 3 and ∼70% survived in water until day 37 (Figure 3E). Chronologically aging *aro7Δ* mutants were more resistant to heat-shock but more sensitive to acetic acid than wild type yeast (Figure 4B and 4E). In the W303-1A background, the deletion of *ARO7* caused an even more severe growth defect and the mutants were short-lived (data not shown). This may depend on a different response to lack of Aro7 in different genetic backgrounds. Notably, extreme growth defects might reflect the inability of old G_0_-arrested cells to reenter the cell cycle to form colonies, simulating a short-lived phenotype.

Of the remaining putative long-lived mutants whose longevity was tested under starvation/extreme CR, *far3Δ*, *far11Δ*, *ppg1Δ*, and *bul1Δ* lived shorter than wild type (the reduction of life span was significant for all the mutants except *bul1Δ*) while *pan2Δ* lived approximately as the wild type (Figure S4A, S4B). Far3 and Far11 are part of a complex that plays a role in promoting G1-arrest in response to pheromone signaling [61]. Notably, Far7, Far8, and Far10 are found in the same protein complex and the corresponding deletion mutants were all identified as putative long-lived strains (Table S6). Furthermore, two of the dubious ORFs whose deletion is associated with longevity extension, *YDR199W* and *YMR052C-A*, overlap with *FAR9*, coding an additional component of the Far complex, and *FAR3*, respectively (Table S6). It is plausible that lack of these proteins may inhibit the G1-arrest triggered by further stimuli, i.e. nutrient shortage, and cause adaptive regrowth. Mutants displaying the adaptive regrowth phenotype may therefore be mistaken for long-lived due to an enrichment of their representation in a pool caused by cell division.

**Figure 8:**
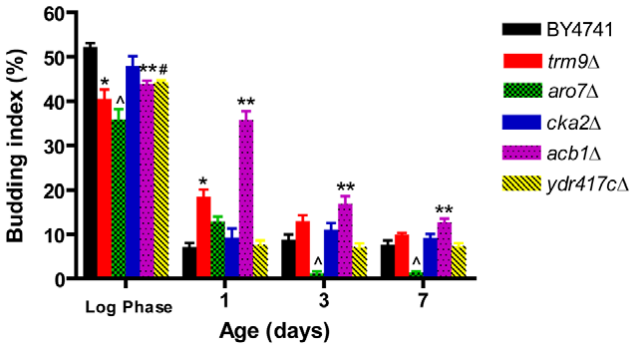
Efficiency of G1/G0-arrest of the novel long-lived mutants. Budding index of BY4741 and mutants lacking either Acb1, Cka2, Trm9, Ydr417c, or Aro7 measured during exponential growth (OD_600_ = 1) and chronological aging on day 1, 3, and 7. Data show mean±SEM. * p<0.01, *trm9Δ vs* WT. **p<0.05, p<0.001, p<0.01, *acb1Δ vs* WT in exponential phase, on day 1, and on day 3–7, respectively. ∧ p<0.001, p<0.01, *aro7Δ* vs WT, in exponential phase, day 7, and on day 3, respectively. # p<0.05, *ydr417cΔ vs* WT. The cultures used were incubated in SDC medium for the duration of the whole experiment.

To further characterize the long-lived mutants identified in this study, we measured the budding index of each of them in exponential phase and during chronological aging (Figure 8). Notably, a more complete G1/G0-arrest, measured as a decrease of budding index, has been observed in chronologically aging long-lived mutants and is believed to contribute to longevity extension via the reduction of replicative stress [40]. Our analysis revealed a lower ratio between budded and unbudded cells in all mutants in comparison with the wild type during the exponential phase. The reduction of budding index was statistically significant for all the mutants except *cka2Δ* in agreement with the mild growth defects observed in the mutants (data not shown). On day 1 the budding index of both *acb1Δ* and *trm9Δ* was significantly higher than that of wild type cells (p<0.01) and in the *acb1Δ* mutant it remained higher until day 7 (p<0.01) (Figure 8). By contrast, the budding index of the *aro7Δ* mutant was significantly lower than that of the wild type on day 3 and 7 (p<0.01) (Figure 8).

## Discussion

The use of the yeast deletion collection combined with a tag microarray detection method has found a wide range of applications, many of which involve drug screening to define their mechanisms of action [62], [63]. Here we adapted this methodology to investigate how different genes affect the chronological life span. By performing a competitive survival assay on a pool of approximately 4800 haploid deletion strains, we identified several novel life span determinants. Analogously to Powers et al., we obtained data supporting the importance of functional mitochondria in long-term survival and identified several autophagy-related genes that are required for normal life span [26] (Figure 2 and Table S7). The autophagic process is down-regulated by the principal pro-aging pathways and work done in yeast, worms, and flies suggests that it is required for longevity extension [46], [48], [64]. Interestingly, we identified a significant number of genes whose deletion is associated with short life span, which are included in the “mitophagy” GO group (GO:0000422) (Table S7). Since in non-dividing cells autophagic breakdown is the only mechanism to remove damaged organelles, we speculate that this is a key element in long-term survival and longevity extension. Furthermore, autophagy plays an important role in the removal of damaged proteins, which are known to accumulate during aging [65].

Because our studies suggest that adaptive regrowth is common in the BY4741 background and also to test the mechanisms of starvation-dependent CLS extension, the longevity tests performed on the individual BY4741 mutants were performed under starvation conditions, whereas the original screen was carried out on cells incubated in medium throughout the experiment. Notably, the great majority of mutations that cause life span extension in medium does so in water [7], [44]. Yeast cultures were transferred to water at day 3, a condition that leads to the activation of an anti-aging response analogous to that promoted by reducing the glucose content of the growth medium and controlled by the same mediators [7], [23], [44]. Thus, our tests on the individual BY4741 mutants studied the effect of individual genes on the starvation/extreme CR-induced longevity extension. In this context, the results shown in Figure 2B indicate that the protein transport to the vacuole is required for the extended life span associated with starvation/extreme CR. However, the dramatic shortening of longevity observed in the *vps27Δ* and *vps25Δ* mutants and their sensitivity to oxidative stress (Figure 2B and 2C) strongly suggest that in chronologically aging yeast protein turnover by autophagy is a crucial function for survival in both regular medium and under starvation/extreme CR. Interestingly, Vps27 and Vps25 are components of the Vps27-Hse1 and ESCRTII complexes, respectively. Both complexes are part of ESCRT system and are involved in the degradation of ubiquitylated transmembrane proteins via the formation of multivesicular bodies (MVBs) [66]. Their key role in survival underlines the importance of plasma membrane and Golgi protein breakdown in preserving cellular function over time.

Of the 14 putative long-lived BY4741 mutants retested, 9 lived longer in water, with 5 of them reaching a significantly extended life span (Figure 3). Three of the latter (*cka2Δ*, *trm9Δ*, and *ydr417cΔ*) were assayed in different genetic backgrounds and their longevity extension phenotype was confirmed in SDC medium. Overall, half of the mutations retested either under starvation/extreme CR or both in medium and under starvation/extreme CR was confirmed to be implicated in life span regulation, underscoring the effectiveness of our experimental approach but also the importance of using the water paradigm to filter out false positives.

Interestingly, none of the long-lived mutations identified here has been identified by the high-throughput analysis performed previously [26]. Similarly, we did not identify any mutants of the TOR/Sch9 or of the other known pro-aging pathway, possibly because mutations in these pathways may affect growth rates and interfere with this growth-based method to determine life span.

In our assay, the interaction between deletion mutants in the same environment might increase the noisiness of the data. In fact, the death of the short-lived mutants and consequently, the release of nutrients, might facilitate the regrowth of other mutants, which in turn might lead to the accumulation of metabolites (e.g. ethanol or acetate) detrimental for cell survival [7], [18]. This death-regrowth dynamic is supported by the increase of CFUs detected on day 12 and 15 of our initial screen (Figure 1A). A certain degree of noise indeed exists in our data. For example, the *trm9Δ* and *ydr417cΔ* mutants were confirmed as long-lived despite the observation that the reproducibility between the two biological replicates as estimated by EDGE analysis did not reach the threshold of q = 0.1 (Table 1). Some noisiness in the data may also explain why none of the genes implicated in yeast apoptosis and known to reduce chronological life span was identified in our screen. Although we do not have results relative to the pro-apoptotic *NDE1* gene (the corresponding mutant was excluded from our dataset, see Materials and Methods), other deletions associated with a down-regulation of yeast apoptosis, e. g. *yca1Δ*, *aif1Δ*, and *ndi1Δ*, were not present in the putative long-lived group generated by K-means clustering. This is not surprising given the modest life span extension caused by the deletion of these genes [67], [68].

Among the long-lived mutants identified is *acb1Δ*. Acb1 is a ∼10kD acyl-CoA-esters binding protein highly conserved in eukaryotes [52]. In yeast, down-regulation of Acb1 activates a stress response, which includes several heat-shock proteins and the cytosolic catalase (Ctt1) [69]. Although in the BY4741 *acb1Δ* mutant we did not observe resistance to H_2_O_2_, the mutant was heat resistant, in agreement with the up-regulation of heat-shock genes [69].

The serine/threonine kinase CK2 is constitutively expressed in all eukaryotes so far investigated and hundreds of CK2 substrates are known. [70]. In yeast, CK2 is required for cell viability, it is primarily located within the nucleus and is implicated in the regulation of chromatin structure and global gene expression [55]. Since in mammalian cells both S6K and Akt/PKB are CK2 substrates [71], [72], it will be informative to test whether Sch9, the closest yeast homologue of these kinases, interacts with CK2 directly or indirectly.

Trm9 is one of several tRNA methylases present in yeast and is conserved in several other species [58]. Lack of Trm9-dependent methylation at U34 is thought to cause the incorporation of the wrong amino acids into proteins [58], which may explain the growth defect observed in the *trm9Δ* mutants. The production of certain defective proteins might: a) simulate a reduction of translation, which is known to extend the life span of *C. elegans* [73] and b) promote chaperone synthesis, which may have an anti-aging role [74].

The deletion of the dubious *YDR417C* ORF, which overlaps with *RPL12b*, causes reduction of growth rate, life span extension under starvation/extreme CR and in regular medium, and heat-resistance. Analogous results were obtained when the deletion of *RPL12b* was analyzed. We have not tested directly whether *YDR417C* or *RPL12b* is responsible for the phenotypes observed. However, since a) in high-throughput gene expression studies no expression corresponding to *YDR417C* has been detected [59], [60] and b) abolishing/reducing the expression of ribosomal proteins causes RLS and CLS extension in yeast and worms, respectively [13], [75], lack of Rpl12b may be the cause of the phenotypes we observed in the *ydr417cΔ* mutant.

The deletion of the aromatic amino acid biosynthetic gene *ARO7* leads to longevity extension and heat-shock resistance in BY4741. This observation points to a general role of amino acid-signaling in life span regulation. In fact, several lines of evidence suggest that the reduction of the amino acid/protein component of the diet might be important for life span extension: 1) removing glutamate or asparagine from the medium promotes yeast CLS extension [26], 2) amino acid-restriction prolongs yeast RLS [76], 3) reduction of the protein content in the diet of *Drosophila* was shown to be the key factor to extend life span [77], 4) in rodents, a simple reduction of either methionine or tryptophan from the diet promotes longevity [78], [79]. It will be important to understand how different amino acids affect life span and whether conserved pathways function to regulate life span in response to amino acid restriction in different species.

It is noteworthy to point out that our screen identified long-lived mutants that are more sensitive to acetic acid than wild type (*trm9Δ*, *ydr417cΔ*, and *aro7Δ*). In this regard, they differ from the long-lived *sch9Δ* and *ras2Δ* mutants, which were shown to be resistant to acetic acid [18] (Figure 4D), suggesting that resistance to high concentrations of this acid is not a requirement for longevity extension. Further experiments are needed to understand whether acetic acid plays a pro-aging role in a *trm9Δ*, *ydr417cΔ*, or *aro7Δ* context.

Notably, the majority of the novel long-lived mutants did not show an increased percent of G1/G0-arrested cells during chronological aging. On the contrary, one of them, *acb1Δ*, showed a significantly higher budding rate up until day 7. Together, these results indicate that, while a tighter G1/G0 arrest may improve chronological survival [18], its role in yeast aging is not central.

In summary, we have identified novel yeast pro-aging genes that point to cell functions previously not linked to life span regulation. Several of these genes are evolutionary conserved suggesting that they may also function to control longevity in other species.

## Materials and Methods

### Yeast strains

Pools of the BY4741 (*MAT*a *his3Δ1*, *leu2Δ0*, *met15Δ0*, *ura3Δ0*) haploid deletion collection were obtained as described previously [35]. All the other strains used for this study were generated in either the DBY746 (*MATα leu2-3*, *112*, *his3Δ*, *trp1-289*, *ura3-52*, *GAL^+^*) or the W303-1A (*MAT*a *leu2-3,112 trp1-1*, *can1-100*, *ura3-1 ade2-1*, *his3-11,15*) genetic backgrounds by one-step gene replacement as described by Brachmann et al. [80]. The complementation tests were performed on the *acb1Δ* mutant after transformation with either a centromeric MoBY plasmid carrying the *ACB1* ORF under its own promoter or a control vector [81].

### Growth conditions and chronological life span assay

Yeast cells were grown in synthetic complete medium (SDC) supplemented with a four-fold excess of tryptophan, leucine, uracil and histidine. Yeast chronological life span was measured as previously described [3]. Briefly, overnight SDC cultures were diluted (1∶200) into fresh SDC medium and incubated until day 3 when no residual cell growth is normally observed. Viability was measured by plating aging cells onto YPD plates and monitoring Colony Forming Units (CFUs) starting from day 3, which was considered to be the initial survival (100%). For starvation/extreme calorie restriction, cells from 3 day-old SDC cultures were washed three times with sterile distilled water and resuspended in water. Every 4–7 days, cells from the water cultures were washed to remove nutrients released from dead cells. To establish significance between the survival of wild type and deletion mutants, the mean life span calculated by curve fitting (Boltzmann sigmoidal) and/or the area under the survival curves were used to perform: 1) one-way ANOVA followed by Tukey's multiple comparison test (Figure 2A, Figure S4A) and 2) paired t-test, two-tailed (rest of the survival studies). The statistical software Prism (GraphPad Software) was used for the analysis.

### TBBt and TBBz treatment

CK2 inhibitors were added to the aging cultures at day 2 and 5 and resistance to heat was tested at day 3. The range of concentrations tested was 10–200 µM for TBBz and 5–15µM for TBBt.

### Budding index measurement

At different time points the budding index of approximately 500 cells was determined visually by using a phase contrast microscope. Prior to cell examination, cell clumps were removed by brief sonication. The data were analyzed by one-way ANOVA followed by Tukey's multiple comparison test.

### Genomic DNA preparation, PCR, and chip hybridization

At day 3, 9, 11, 15, and 20, samples from the aging pools were diluted in fresh medium to an initial density of 6.25×10^5^/mL and incubated at 30°C until the cultures reached a density of 10^7^/mL. Approximately 2×10^7^ cells were spun down and cell pellets were stored at −20°C until further processing. Genomic DNA was extracted using the YeaStar Genomic DNA kit (Zymo Research) as previously described [35]. TAG hybridization and processing of microarrays were performed as described by Pierce et al. [35].

### Data analysis

Barcode probe intensities were extracted and processed as described previously [35]. For each time point, the corresponding array was mean normalized and log_2_ ratios were calculated with respect to the day 3 time point to obtain mutant-specific aging profiles (Table S2). Approximately 500 mutants whose tag intensity was similar to the background at each time point (e. g. mutants growing extremely slowly or mutants whose tags hybridize poorly) were excluded from the analysis. Next, K-means clustering (K = 10) was performed to identify strains with similar aging profiles. Profiles for each strain were averaged between replicates prior to K-means clustering. The root squared mean errors (RSME) between the two replicates were calculated and 413 mutants with high RSME (90^th^ percentile) were also excluded from the analysis (Table S2). Clusters were classified as short-lived and long-lived by manual inspection. A novel significance method was recently developed for identifying differentially expressed genes in longitudinal time course microarray studies [42]. We adapted this method to our aging dataset, which is analogous to a time course microarray as it involves repeated sampling and measuring from a pool of mutants. This analysis was performed using the EDGE analysis software package [43] to identify strains whose representation in the pool over the course of the experiment changed consistently in both replicate experiments. The time course differential expression analysis option (‘within class’ analysis) was used. Using a q-value cutoff of 0.1, we identified 438 strains that were significantly overrepresented or underrepresented in the pool over the course of the experiment. The q-value estimates the false-discovery rate when calling a gene significant [82], [83]. The survival curves for each individual mutant reported on Table S3 were generated by multiplying the fold change in the microarray intensities (Table S2) by the CFUs of the pool at each time point (Table S1).

### Gene ontology analysis

The short-lived mutants identified by K-means clustering were subjected to Gene ontology enrichment analysis. Gene ontology analysis was performed using the Gene Ontology (GO) Term Finder (http://go.princeton.edu) [84], which uses a hypergeometric distribution to determine whether GO terms are enriched in a list of genes at a frequency greater than that expected by chance.

### Stress resistance assays

Heat shock resistance was measured by spotting serial dilutions (10-fold dilutions, starting from a cell density of ∼10^8^/mL) of cells removed from day 1 or 3 SDC cultures onto YPD plates and incubating at either 55°C (heat-shocked) or 30°C (control) for 60–240 min. After the heat-shock, plates were transferred to 30°C and incubated for 2–3 days. For the oxidative stress resistance assay, cells were diluted to a cell density of 10^7^/mL in K-phosphate buffer, pH6.0, and treated with 100–200 mM of hydrogen peroxide for 30 minutes. Serially diluted (10-fold) control and treated cells were spotted onto YPD plates and incubated at 30°C for 2–3 days. For acetic acid resistance, day 3–5 cultures (0.5 mL) were treated with 300–500 mM acetic acid for 180 min. After the treatment, serially diluted cells were spotted onto YDP plates and incubated at 30°C for 2–3 days. The pH of the acetic acid-treated cultures was ∼3 and did not differ depending on the mutant, neither it changed depending on the acetic acid concentration used. All experiments were repeated 2–3 times with similar results.

All supplementary data can also be downloaded from our webpage: http://chemogenomics.med.utoronto.ca/supplemental/lifespan/

### Note added during the production process

While this article was being revised, Metecic M et al. published a paper describing a screen for long- and short-lived mutants similar to the one reported here [Matecic M, Smith DL, Pan X, Maqani N, Bekiranov S, et al. (2010) A microarray-based genetic screen for yeast chronological aging factors. PLoS Genet 6: e1000921. doi:10.1371/journal.pgen.1000921].

## Supporting Information

Figure S1 CLS of wild type BY4741 and *cup9Δ* mutants switched to water on day 3. A representative experiment is shown.
(0.12 MB TIF)

Figure S2 CLS of BY4741 and *acb1Δ* transformed with either control vector or centromeric plasmid carrying the *ACB1* gene driven by its own promoter. Yeast strains were grown in either SDC or in selective SDC-uracil and transferred to water on day 3. A representative experiment performed in triplicate is shown.
(0.16 MB TIF)

Figure S3 (A) Heat-shock resistance of day 3 DBY746 cultures treated with TBBt (5–15 µM on day 2). (B) CLS of DBY746 cells treated with TBBt on day 2 and 5. DMSO was used as a vehicle. A representative experiment is shown.
(0.72 MB TIF)

Figure S4 CLS of putative long-lived mutants identified by genome-wide screen. (A) CLS of wild type (BY4741) and mutants lacking Far3, Far11, Pan2, or Bul1 transferred to water on day 3. A representative experiment performed in triplicate is shown. B) CLS of wild type and of a *ppg1Δ* mutant under starvation/extreme CR. The average of two independent experiments performed in duplicate is shown.
(0.30 MB TIF)

Table S1 CFUs values relative to the survival of the two YKO pools used for the genome-wide screen.
(0.02 MB XLS)

Table S2 Mutant-specific aging profiles were obtained by calculating the log_2_ ratios for each time point using day 3 as a reference (see Materials and Methods). Data from both biological replicates and their average are shown. The root squared mean error (RSME) between the two replicates is also shown. The last column on the right indicates whether the corresponding mutants were included in the K-means clustering analysis.
(1.35 MB XLS)

Table S3 The CFUs for each strain for pool 1 and 2 at each time point were estimated by multiplying the microarray fold-ratio for each strain (Table S2) by the CFUs number for the pools at the corresponding time point (Table S1). The average number of CFUs was estimated by averaging between the two pools.
(0.76 MB XLS)

Table S4 Deletion mutants were ranked by p and q values obtained by EDGE analysis.
(0.42 MB XLS)

Table S5 p and q values obtained by EDGE analysis for the mutants classified as short-lived by K-means clustering.
(0.07 MB XLS)

Table S6 p and q values obtained by EDGE analysis for the mutants classified as long-lived by K-means clustering.
(0.02 MB XLS)

Table S7 Gene ontology analysis of the short-lived mutants identified by K-means clustering. Significantly enriched categories (p<0.01) are enlisted.
(0.08 MB XLS)
