# Supplementary figures and images for: Genome-Wide Screen in Saccharomyces cerevisiae Identifies Vacuolar Protein Sorting, Autophagy, Biosynthetic, and tRNA Methylation Genes Involved in Life Span Regulation
Source: PLoS Genet. 2010 Jul 15;6(7):e1001024. doi: 10.1371/journal.pgen.1001024 (PMC2904796; doi:10.1371/journal.pgen.1001024)

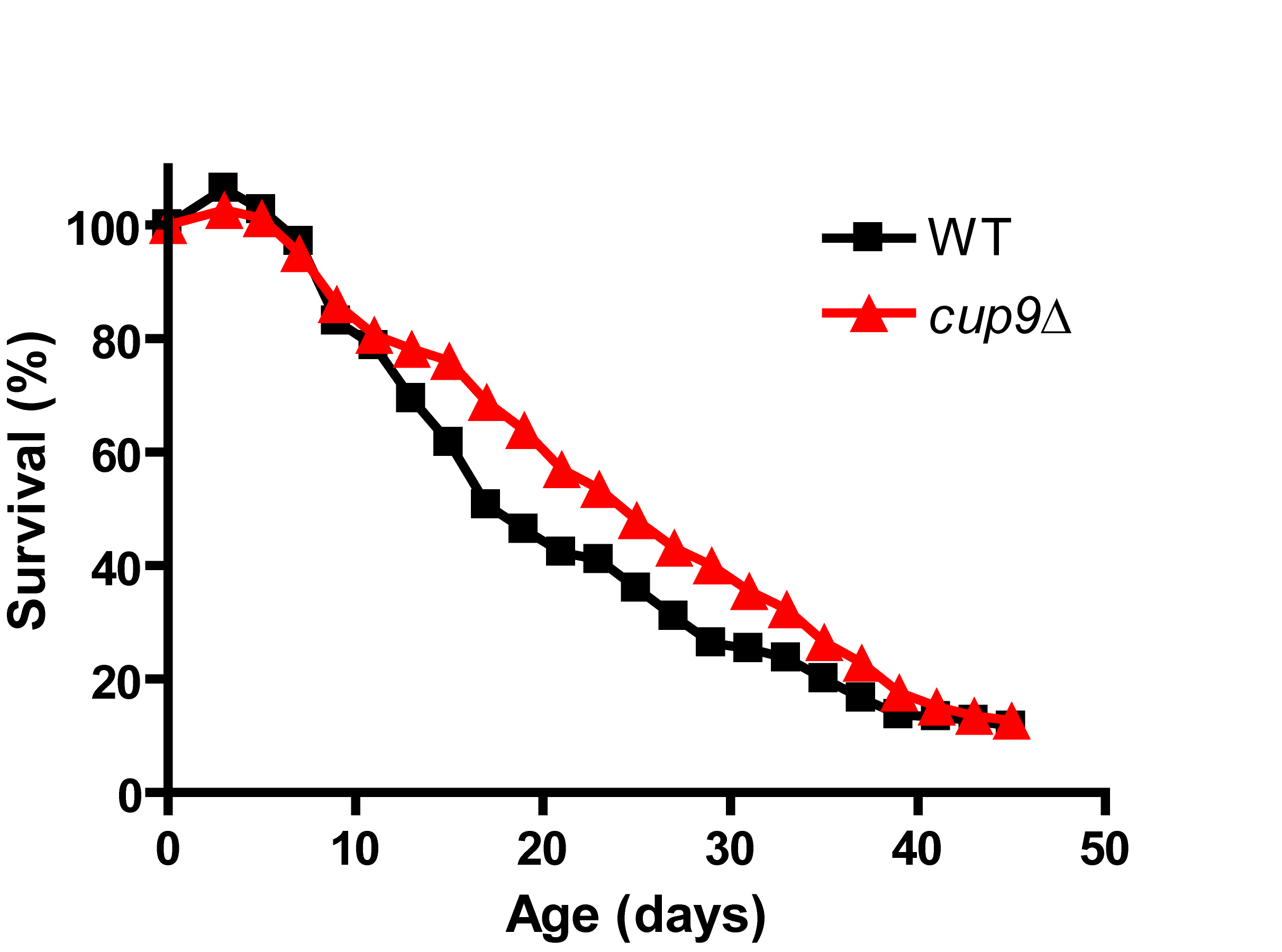

Supplement: Figure S1 — CLS of wild type BY4741 and cup9Δ mutants switched to water on day 3. A representative experiment is shown. (0.12 MB TIF) [file pgen.1001024.s001.tif]

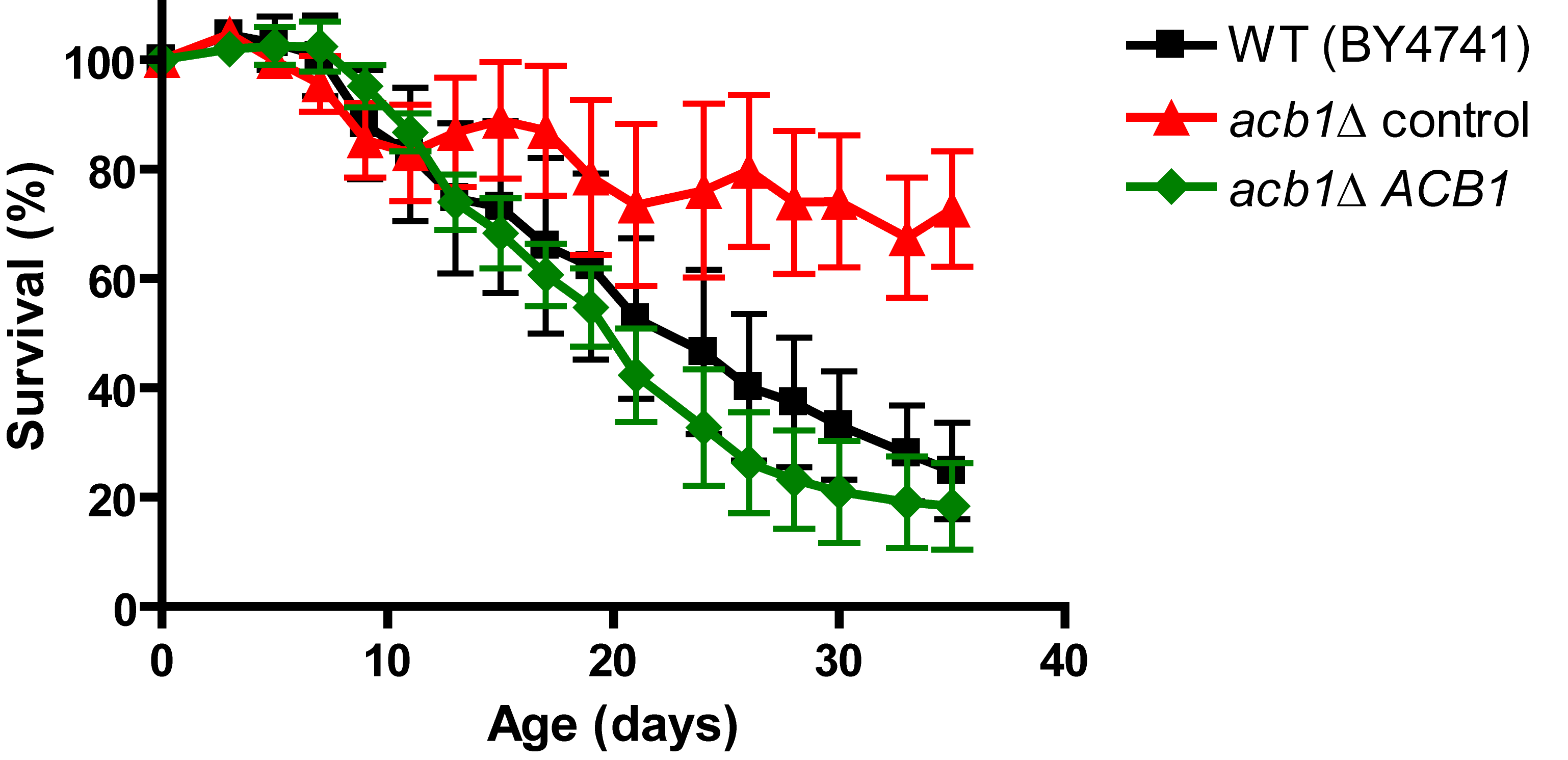

Supplement: Figure S2 — CLS of BY4741 and acb1Δ transformed with either control vector or centromeric plasmid carrying the ACB1 gene driven by its own promoter. Yeast strains were grown in either SDC or in selective SDC-uracil and transferred to water on day 3. A representative experiment performed in triplicate is shown. (0.16 MB TIF) [file pgen.1001024.s002.tif]

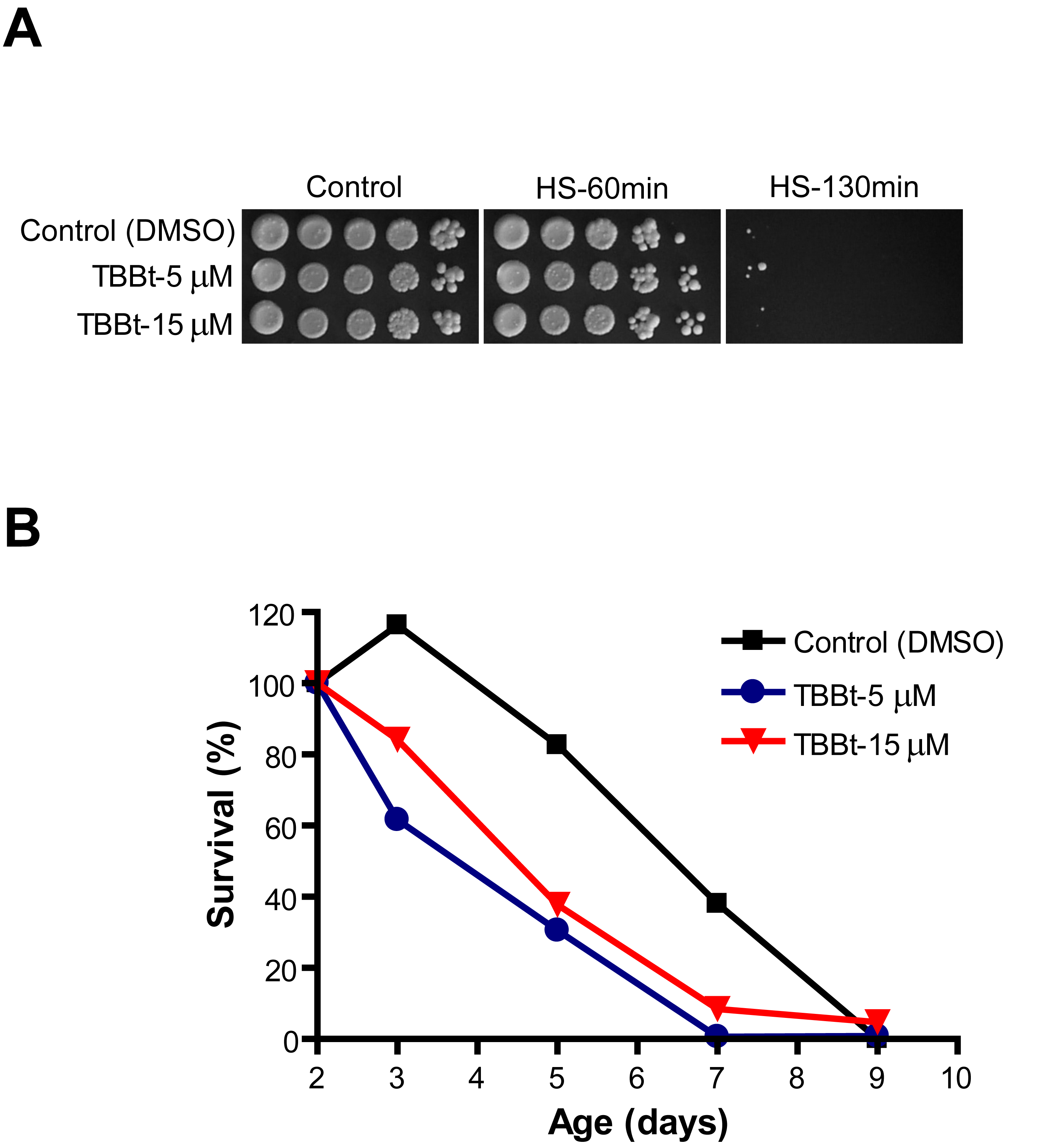

Supplement: Figure S3 — (A) Heat-shock resistance of day 3 DBY746 cultures treated with TBBt (5–15 µM on day 2). (B) CLS of DBY746 cells treated with TBBt on day 2 and 5. DMSO was used as a vehicle. A representative experiment is shown. (0.72 MB TIF) [file pgen.1001024.s003.tif]

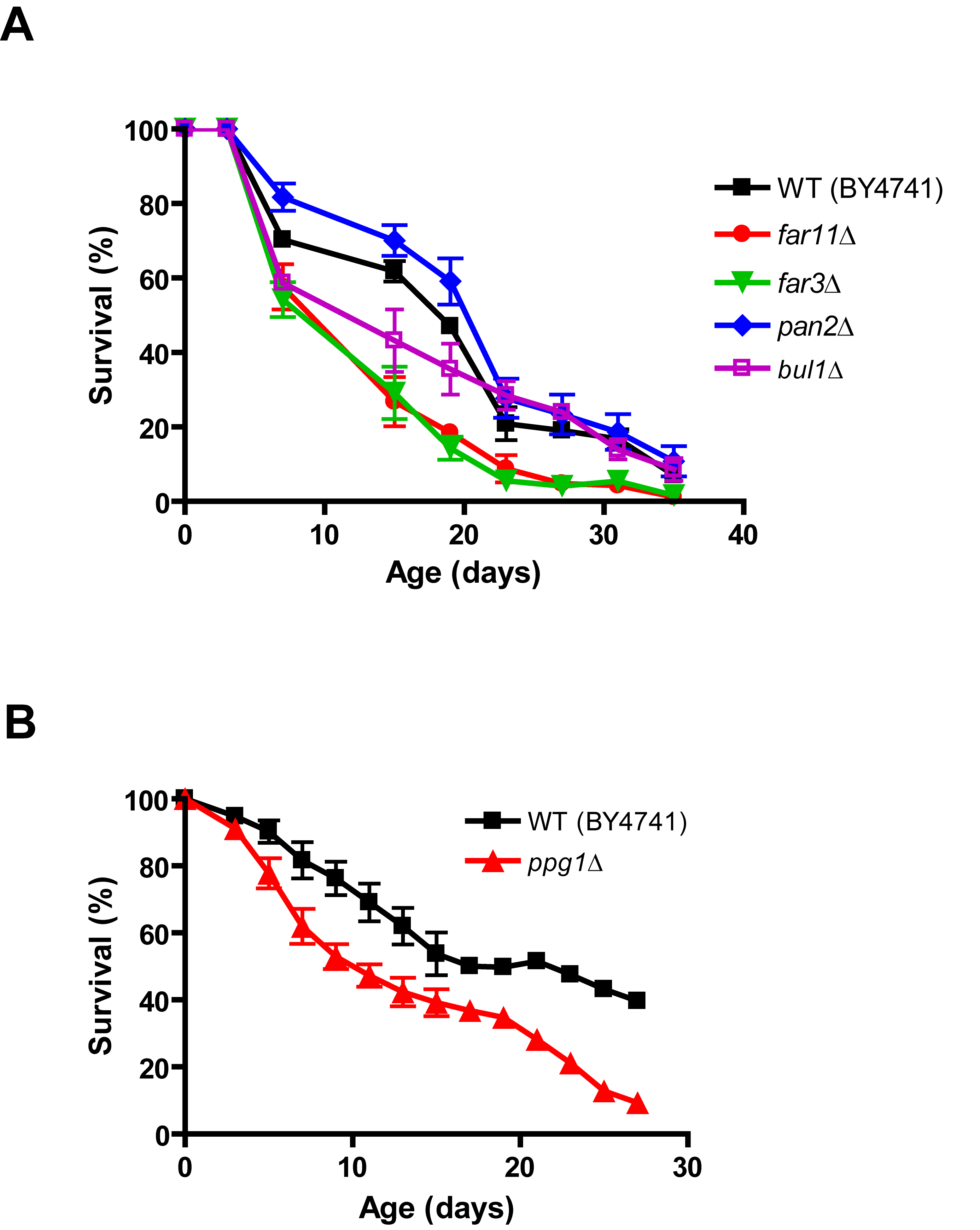

Supplement: Figure S4 — CLS of putative long-lived mutants identified by genome-wide screen. (A) CLS of wild type (BY4741) and mutants lacking Far3, Far11, Pan2, or Bul1 transferred to water on day 3. A representative experiment performed in triplicate is shown. B) CLS of wild type and of a ppg1Δ mutant under starvation/extreme CR. The average of two independent experiments performed in duplicate is shown. (0.30 MB TIF) [file pgen.1001024.s004.tif]
